# Relationship between Maternal Mood Disorders and Dietary Intake of 3-Year-Olds

**DOI:** 10.1155/2021/5597836

**Published:** 2021-12-17

**Authors:** Minatsu Kobayashi, Kohei Ogawa, Naho Morisaki, Hisako Tanaka, Reiko Horikawa, Kevin Y. Urayama

**Affiliations:** ^1^Department of Food Science, Faculty of Home Economics, Otsuma Women's University, Tokyo, Japan; ^2^Center for Maternal-Fetal,Neonatal and Reproductive Medicine, National Center for Child Health and Development, Tokyo, Japan; ^3^Department of Social Medicine, National Center for Child Health and Development, Tokyo, Japan; ^4^Division of Endocrinology and Metabolism, National Center for Child Health and Development, Tokyo, Japan; ^5^Graduate School of Public Health, St. Luke's International University, Tokyo, Japan

## Abstract

Maternal depression affects parenting and children's early development, but its effect on dietary intake is unknown. While husbands' involvement in parenting and having friends to talk to may reduce childcare stress, this has not been thoroughly studied. In this study, mothers were stratified by the presence or absence of mood disorders, and the effects of support from their husbands and friends on the dietary intake of their 3-year-old children were examined. This cross-sectional analysis included 920 mother-child pairs examined at the National Center for Child Health and Development in Japan. Dietary intake was assessed using a brief dietary history questionnaire, and physical measurements were taken when the children were 3 years old. The Kessler Psychological Distress Scale was used to screen for maternal mood disorders, 3 years after delivery. The presence or absence of the husband's assistance with housework and childcare, mental support, and friends was obtained from a self-administered questionnaire when the child was 3 years old. Differences in the children's physical measurements, energy, and food intake with the presence or absence of support for subjects with or without mood disorders were compared. Mothers with support from husbands or friends had significantly fewer mood disorders. Support from friends and family did not affect the children's physical development and whether or not mothers had mood disorder symptoms. However, children's vegetable intake was higher if mothers were supported. Children of mothers with mood disorders had a significantly higher vegetable intake and fruit intake, depending on the support from friends (*P*=0.046,  *P*=0.037); thus, such support may increase children's vegetable and fruit intake. The results of this study revealed the importance of supportive friends and family regarding childcare.

## 1. Introduction

Since the birth rate plummeted in Western countries in the 1970s, future population declines have been predicted. Birth rates continue to fall, not only in developed countries but also in developing countries [1]. The total fertility rate in Japan has been below 2.0 since 1975, and the increase in the ratio of the elderly population (aged 65 years and older) to the productive age population (15–64 years) makes it difficult to maintain social security systems, including pensions [2, 3]. To address the issue of declining birth rates in Japan, the Ministry of Health, Labor, and Welfare has promoted various measures such as the improvement of childcare services, consultation, and support systems for childrearing [4]. However, according to the Cabinet Office's survey on measures to cope with declining birthrates, 46.7% of women in the younger generation are anxious about raising children, which was 14.2% higher than men in the same generation [5].

There was no improvement in mothers' subjective childrearing anxiety in the “Healthy Japanese 21 survey,” which shows the direction and goals of child health care in the twenty-first century [6]. Unless mothers' anxieties about childcare are alleviated, it will be difficult to solve the problem of declining birth rates. In situations where mothers find it difficult to raise children, cooperation of family members, especially husbands who show understanding and have cooperative attitudes, is largely related to decreasing maternal stress [7]. Mothers and fathers in the high-scoring group for childrearing stress are reported to have significantly fewer conversations regarding parenting in the home [3]. In addition, parenting-related stress that mothers experience daily is reported to be related to the severity of maternal depressive symptoms [8]. It has been pointed out that prolonged depression and depressed mood in mothers may lead to anxiety, difficulty in raising children, abandonment of children, and, in the worst cases, abuse of children [7]. It has also been reported that maternal depression affects parenting behavior, including dietary parenting, which may affect children's early development [9, 10]. Children's diets affect not only their physical development but also their dietary habits throughout the growing process. They also cultivate basic lifestyle practices, ensuring that they lead a healthy life. However, there are no further reports on the relationship between maternal depression or mood disorders and children's dietary intake status.

It is necessary to reduce the difficulty involved with childrearing by providing support to mothers with depression. However, it is often difficult to rely on families and relatives, because of geographical and temporal factors, especially in urban areas. In fact, 55.9% of all households in Japan have nuclear families [1]. The involvement of husbands and partners has been reported to reduce the difficulty of childrearing [11], according to the Basic Survey on Social Life by the Ministry of Internal Affairs and Communications. However, when comparing the childcare time of husbands and wives with children under 6 years old, the time spent raising children was 3.45 hours for wives and only 0.49 hours for husbands [12]. There is also a report to the effect that it is possible to reduce childcare stress by having friends with whom to exchange childrearing information [13]. Therefore, in this study, the effects of support from husbands and friends on the dietary intake of 3-year-old children were examined after mothers were stratified by mood disorders.

It is possible to present a system of support for mothers who find it difficult to raise children by stratifying and analyzing them according to the presence or absence of mood disorders.

## 2. Materials and Methods

### 2.1. Study Population

Our study was performed at the National Center for Child Health and Development (NCCHD) in suburban Tokyo, Japan. Enrollment of pregnant women in the cohort was conducted from May 13, 2010, to November 28, 2013. Participants were recruited during their first antenatal visits, which usually take place in gestational weeks 6–14. A survey of the mothers' mood disorders, the children's food intake, and anthropometric measurements for children were assessed when a health checkup at age 3 was conducted in the hospital.

Of the 2,309 women who provided written informed consent at their antenatal visits, 1,951 submitted their second informed consent to continue participating in the postpartum cohort. Among these respondents, 1,041 answered a questionnaire when their offspring was 3 years of age during the medical checkup. The following exclusions were made: 52 multiple pregnancies, 20 mothers who had mental disorders before pregnancy, 23 mothers with no information regarding the availability of support from husbands or friends, 8 children aged over 4 years or under 2 years and 6 months, and 18 children who reported extreme energy intake (1.0% at the top and bottom). In total, 920 sets of mother and child data were included in the analysis.

The study protocol was approved by the Institutional Review Board at the National Center for Child Health and Development on August 2, 2010 (project number 417).

### 2.2. Assessment of Children's Anthropometric Measurements

Physical measurements at birth and at the age of 3 years were performed at the NCCHD. To assess children's growth, the Kaup index (calculated by weight (kg)/(height (cm))^2^ × 10^4^) was used as an indicator of the development of children aged 3–5 years.

### 2.3. Assessment of Children's Dietary Intake

The children's dietary food intake was assessed using a brief self-administered diet-history questionnaire for use in children (BDHQ3y) by their mothers. The BDHQ3y was developed for assessment of dietary intake during the month preceding the study in Japanese preschool children aged 3–6 years. The BDHQ3y included 66 food items and nonalcoholic beverage items and asked participants about the habitual consumption of the listed foods within a one-month period [14]. Validation of the BDHQ3y in computing total energy, nutrients, and food groups was assessed using the 3-day dietary records of 61 children aged 3-4 years. It has been reported that the BDHQ3y is a good candidate for dietary intake assessment in Japanese preschool children [14]. We calculated 66 food intakes and categorized 14 food groups. The total energy intake per day was calculated using a food composition table developed for the BDHQ3y, based on the Standardized Tables of Food Composition in Japan (2015 edition) [15]. Food intake was adjusted by total energy intake using a residual model [16].

### 2.4. Assessment of the Mothers' Basic Information

The following maternal sociodemographic data were collected using the distributed questionnaires during registration: marital status (married, not married), household income (<4 million yen, 4–8 million yen, >8 million yen, missing), and educational history (training school, high school, or less, graduated college, graduated university, missing). Additionally, when the child was 3 years old, the following information was collected: working status (full-time, part-time, not working), current smoking status (yes, no), and drinking habits of at least once a week (yes, no). The mother's age, height, and weight were retrieved from their medical charts, and their body mass index (BMI) was calculated based on their height and weight. Other baseline characteristics from the medical records were also included and categorized as follows: the presence of mental illness before pregnancy (yes, no), presently receiving treatment for a disease (yes, no), and experience of parity (yes, no). The data obtained at the time of the mother's initial enrollment and the data obtained three years later were distinguished and rewritten. The data of postpartum depression one month after delivery was obtained with the Edinburgh Postnatal Depression Scale (EPDS). The presence or absence of assistance with housework or emotional assistance from the husband and the presence or absence of friends with whom they could talk about childcare were obtained using a self-administered questionnaire completed by the mother 3 years after giving birth.

### 2.5. Measurement of the Mothers' Mood Disorders

A version of the Kessler Psychological Distress Scale (K-6) translated into Japanese was used to screen women for mood disorders 3 years after delivery. The K-6 is a 6-item self-report screening tool for mood disorders. Responses are recorded using a five-point Likert-type scale (0 = all of the time, 1 = most of the time, 2 = some of the time, 3 = a little of the time, and 4 = none of the time). Total scores can range from 0 to 24, and the cut-off value of ≥10 has been shown to indicate good sensitivity and specificity for Japanese women [17].

### 2.6. Statistical Analysis

Initially, the mothers' basic information data were expressed as mean ± standard deviation [18] for continuous variables and a percentage for categorical variables. Data on the presence or absence of any support were expressed as percentages. Differences in continuous variables between subjects with and without mood disorders were tested using Student's *t*-test. Differences in categorical variables between subjects with and without a mood disorder were tested using the chi-square test whenever the expected values in any of the cells of a contingency table were above five, while Fisher's exact test was used for those cells below five. After dividing the subjects into those with and without mood disorders, and in examining differences in children's anthropometric measurements and dietary intake in each group depending on the presence or absence of support from a husband or friend, an analysis of covariance was performed. Adjustments were made for possible confounding factors related to maternal mood disorder, including the sex and age of the child. All statistical analyses were conducted using SAS statistical software ver. 9.4. (SAS Institute Inc.), and a two-sided *P* value of <0.05 was considered statistically significant when testing the hypothesis.

## 3. Results

There were no marked differences in maternal age, height, weight, and BMI and in the children's age, height, and weight, based on the presence or absence of maternal mood disorders. Additionally, differences were not observed by marital status, treatment status, smoking status, drinking habits, household income, education history, birth history, and child's gender. Mothers with mood disorders had a significantly higher incidence of postpartum depression (*P* < 0.001) and were more likely to be part-time workers (*P*=0.053) (Table 1).

The presence of mood disorders was significantly lower for mothers whose husbands helped with household chores, parenting support, and mental support and who had friends with whom to talk about parenting (*P* < 0.0001) (Table 2).

There were no significant differences in height, weight, and Kaup index of children, even if their mother had a mood disorder, whether there was a presence or absence of assistance with housework or mental assistance by the husband, and whether there was a presence or absence of friends with whom to talk about parenting (Table 3).

The 3-year-old children of mothers—both with and without mood disorders—who received assistance with housework from their husbands had a higher vegetable intake (10 g or more) than those who did not, although the difference was not significant. There were no differences in energy intake or other food intake based on the presence or absence of assistance with housework by the husband (data not shown).

In mothers without mood disorders, the vegetable intake of their children was 12.1 g higher, when they received emotional support from their husbands (*P*=0.052); in mothers with mood disorders, the vegetable intake of their children was 9.5 g higher, when they received emotional support from their husbands. However, it was not statistically significant. There was no difference in the energy intake or other food intake of children, depending on the presence or absence of mental assistance by the husband (Table 4).

In mothers without mood disorders, children had lower sugar intake (*P*=0.012) and higher fat and oil intake (*P*=0.032) if the mothers had friends with whom they could discuss parenting. Additionally, children's vegetable intake was 13.4 g higher if their mothers had friends with whom they could discuss parenting (*P*=0.033). In mothers with mood disorders, the children's energy intake was higher (*P*=0.029), vegetable intake was 16.9 g higher (*P*=0.046), fruit intake was 16.8 g higher (*P*=0.037), and cereal intake was 28.0 g lower (*P*=0.001) if the mothers had friends to talk to about parenting (Table 5).

## 4. Discussion

This study found that mothers with mood disorders had less childcare support than those without. Support for mothers by family and friends was associated with increased vegetable intake in their children, with or without the mothers having a mood disorder. It was found that children's vegetable intake was significantly different depending on the presence or absence of friends with whom the mother could consult.

In this study, we divided the subjects into two groups according to the presence or absence of mood disorders, using the K-6, and examined the effects of child growth with and without childcare support for mothers in each group. Previous studies have demonstrated that excessive stress and mental disorders in mothers, as well as childcare support for mothers with mental disorders, affect children's growth and development [19, 20]. In this study, we focused only on children's physical development but did not find any difference in height, weight, or Kaup index between children of mothers with and without mood disorders, depending on the presence or absence of childcare support. Most of the subjects of this study are economically fortunate people; these results may differ from results reported in studies involving low-income populations. Longitudinal observation is necessary, considering the effects of prolonged parenting stress on a child's growth and development.

Although it has been reported that the presence of maternal mental health problems negatively affects children's nutritional intake [21], studies have not investigated the effect of mothers with mental health problems receiving childcare support on their children's diet. According to the results of the present study, when mothers, both with and without mood disorders, received support from friends, their children's vegetable intake was significantly higher. When mothers, both with and without mood disorders, received mental support from their husbands, their children's vegetable intake was higher. In addition, when mothers with mood disorders received support from friends, their children's fruit intake was significantly higher.

It is expected that clearer conclusions will be drawn by increasing the sample sizes in the future. For children in early childhood to eat vegetables, it is necessary to devise ways to make them easier to eat. It is possible that the mother's ability to do so, with the support of her husband or partner, may affect children's vegetable intake. It has been reported that mothers' parenting stress is reduced if their husbands help with household chores and provide them with emotional support [22]. Interestingly, the present study also demonstrated that having a friend with whom mothers can talk about childcare has a more significant effect on their children's vegetable and fruit intake, compared to mothers receiving support from their husbands. This is an important finding, as eating enough vegetables and fruits reduces the risk of future noncommunicable diseases (e.g., cardiovascular disease).

The impact on children's diets, other than vegetable and fruit intake, was largely dependent on the presence or absence of friends. From the results of the Cabinet Office's Awareness Survey on Measures for the Declining Birthrate, the most important factor to support childrearing in the community was “there are people and places where you can feel free to consult about your concerns on childrearing” [5]. If mothers with mental disorders received support from friends, their children's total energy intake was significantly higher and cereal intake was significantly lower. A large amount of cereal that contributes to energy intake leads to a diet with low nutrient density, which has a very harmful effect on developing children. In the case of low-income households and mothers who lack nutrition knowledge, cereal intake tends to be high and vegetable intake tends to be low [18, 20]. There is no report showing that the presence or absence of support from friends affects energy intake, cereal intake, fruit intake, and vegetable intake in subjects with a high annual income, as in this study. It is possible that mothers who do not have friends with whom to talk are less active in raising their children and tend to prepare simple meals, which causes low vegetable intake and high cereal intake. Early childhood represents an important time for establishing dietary habits that follow through into adulthood. Considering the importance of the healthy growth of children after the age of 3 years, it is important that mothers have friends with whom they can consult about raising children. However, if the mother works full-time, there is concern that they may not have enough time to prepare meals and may only be able to prepare simple meals. Therefore, we stratified mothers with mood disorders according to their employment status and compared the difference in food intake between those with and without support from friends. Although the number of subjects working full-time was small and not statistically significant, children of mothers who worked full-time tended to consume 32.3 g more vegetables when their mothers had the support of a friend (Supplementary Table 1).

The strength of this study is that all subjects were continuously surveyed at the hospital where they gave birth, and data collection and the timing of the survey were well managed; therefore, patients with prenatal mental disorders were adequately excluded. Moreover, the anthropometric measurement values of 3-year-old children were measured at the same hospital at the time of the outpatient examination, and the reliability of the measured inspection values should be high. The dietary survey of 3-year-old children was evaluated using the validated BDHQ3y.

However, most of the subjects of this study were highly educated and came from high-income households; therefore, none of the households were extremely malnourished. The absence of children with extreme malnourishment means that there are few variations in child development and nutritional status. However, in this study, it was clarified that even in subjects with small variations in nutritional status, there is a difference in the intake of vegetables, with and without childcare support. Moreover, since this was a cross-sectional evaluation, the duration of maternal mood disorder cannot be determined. We divided mothers with mood disorders into groups based on their history of postpartum depression (EPDS score ≥9), and child dietary intake was compared by their mothers' group and depended on whether they had friends with whom they could talk about parenting. We found that among the mothers, both with and without a history of postpartum depression, the children of those who had friends with whom they could discuss parenting had a significantly lower intake of cereals and, although not statistically significant, a higher intake of vegetables and fruits (Supplementary Table 2). It was not possible to distinguish between those who had postpartum depression and mood disorder symptoms continuously up to 3 years after delivery and those who had newly developed mood disorders 3 years after delivery.

## 5. Conclusions

This study showed that childcare support being available to the mother did not affect the child's physical development but did affect their diet, whether or not the mothers had mood disorder symptoms. This study found that there was a greater impact on those who had a greater presence of friends with whom they could consult than those who had only their husbands' support. With today's ever-increasing number of nuclear families, not only family support but also community participation in sharing childcare is expected to relieve mothers' childcare stress and contribute to children's healthy growth.

## Figures and Tables

**Table 1:** Characteristics of mothers, both with and without mood disorders (K-6 score ≥10), and their children three years after childbirth.

		Without mood disorders	With mood disorders	*P* value^a^
		(*n* = 559)	(*n* = 361)	
		(mean ± SE)	(mean ± SE)	

Maternal age	Year	39.6 ± 0.2	39.3 ± 0.2	0.182
Maternal height	cm	159.3 ± 0.2	159.0 ± 0.3	0.339
Maternal weight	kg	53.0 ± 0.3	52.2 ± 0.4	0.117
Maternal BMI		20.9 ± 0.1	20.6 ± 0.1	0.229
Child's age	y	3.0 ± 0.0	3.0 ± 0.0	0.419
Child's birth height	cm	49.3 ± 0.1	49.0 ± 0.1	0.198
Child's birth weight	g	3001.6 ± 17.8	2977.8 ± 23.8	0.415
		(%)	(%)	
Marital status				
Married		99.1	99.1	0.911
Not married		0.9	0.9	
Postpartum depression^c^				
Yes		10.9	26.0	<0.001
No		89.1	74.0	
Under continuous treatment^d^				
Yes		23.5	28.2	0.114
No		76.5	71.8	
Employment status				
Full-time worker		14.0	12.0	0.053
Part-time worker		13.4	19.3	
Not working		72.6	68.6	
Currently smoking				
Yes		2.3	2.5	0.879
No		97.7	97.5	
Drinking habit^e^				
Yes		29.6	30.4	0.793
No		70.5	69.6	
Annual income (yen)				
Less than 4 million		5.0	8.0	0.297
4 to 8 million		31.3	31.6	
Over 8 million		55.1	51.8	
Missing		8.6	8.6	
Maternal education				
Training school, high school, or less		6.1	8.3	0.573
Graduated college		30.0	29.6	
Graduated university		60.1	57.9	
Missing		4.8	4.2	
Experience of giving birth				
Yes		33.8	36.6	0.392
Sex of the child				
Male		53.5	50.7	0.407

^a^ *P* value was calculated as Student's *t*-test or Chi-squared test. ^b^*P* value was calculated as Fisher's exact test. ^c^EPDS score ≥9. ^d^There are ongoing diseases under treatment. ^e^More than once a week.

**Table 2:** Presence of support for mothers with and without mood disorders three years after childbirth.

	Without mood disorders	With mood disorders	*P* value^a^
	(*n* = 559)	(*n* = 361)	
	*n* (%)	*n* (%)	
Husbands help with housework and childcare			
Not at all	6 (1.1)	15 (4.2)	<0.0001
Not often	46 (8.2)	55 (15.2)	
A little	169 (30.2)	136 (37.7)	
Often	338 (60.5)	155 (42.9)	
Husbands provide emotional support			
Not at all	12 (2.2)	25 (6.9)	<0.0001
Not often	22 (3.9)	54 (15.0)	
A little	176 (31.5)	124 (34.4)	
Often	349 (62.4)	158 (43.8)	
Have friends with whom they can talk about childbirth and childcare			
Not at all	2 (0.4)	7 (1.9)	<0.0001
Not often	19 (3.4)	30 (8.3)	
A little	181 (32.4)	166 (46.0)	
Often	357 (63.9)	158 (43.8)	

^a^ *P* value was calculated using the Chi-squared test.

**Table 3:** Developmental status of children of mothers, with and without mood disorders, and of those with and without support three years after childbirth.

	Without mood disorders		*P* value^b^	With mood disorders		*P* value^b^
	With support	Without support		With support	Without support	
	(mean ± SE)	(mean ± SE)		(mean ± SE)	(mean ± SE)	
*Husbands help with housework and childcare*						
*n*	338	221		155	206	
Child's height (cm)	92.5 ± 0.2	92.1 ± 0.3	0.234	92.2 ± 0.3	92.4 ± 0.3	0.665
Child's weight (kg)	13.7 ± 0.1	13.5 ± 0.1	0.324	13.4 ± 0.1	13.4 ± 0.1	0.870
Child's Kaup index	16.0 ± 0.1	15.9 ± 0.1	0.729	15.7 ± 0.1	15.7 ± 0.1	0.884

*Husbands provide emotional support*						
*n*	349	210		158	203	
Child's height (cm)	92.5 ± 0.2	92.2 ± 0.3	0.357	92.4 ± 0.3	92.3 ± 0.3	0.814
Child's weight (kg)	13.6 ± 0.1	13.7 ± 0.1	0.779	13.5 ± 0.1	13.4 ± 0.1	0.418
Child's Kaup index	15.9 ± 0.1	16.0 ± 0.1	0.294	15.8 ± 0.1	15.7 ± 0.1	0.443

*Have friends with whom they can talk about childbirth and childcare*						
*n*	357	202		158	203	
Child's height (cm)	92.4 ± 0.2	92.3 ± 0.3	0.679	92.0 ± 0.3	92.6 ± 0.3	0.151
Child's weight (kg)	13.6 ± 0.1	13.7 ± 0.1	0.793	13.4 ± 0.1	13.5 ± 0.1	0.404
Child's Kaup index	15.9 ± 0.1	16.0 ± 0.1	0.430	15.8 ± 0.1	15.7 ± 0.1	0.631

^a^K-6 score ≥10. ^b^*P* value was calculated as ANCOVA with the child's sex and age as covariates.

**Table 4:** Energy and food intake of three-year-old children of mothers, with and without mood disorders, and of those with and without emotional support from their husband and partner.

(g)	Without mood disorders (*n* = 559)		*P* value^b^	With mood disorders (*n* = 361)		*P* value^b^
	With support	Without support		With support	Without support	
	(*n* = 349)	(*n* = 210)		(*n* = 158)	(*n* = 203)	
	(mean ± SE)	(mean ± SE)		(mean ± SE)	(mean ± SE)	
Energy (kcal)	1401.0 ± 18.4	1396.1 ± 23.7	0.871	1417.8 ± 25.7	1383.5 ± 22.7	0.317
Cereals	308.2 ± 4.2	303.1 ± 5.5	0.464	311.1 ± 6.2	310.8 ± 5.5	0.970
Potatoes and starches	22.7 ± 0.8	23.0 ± 1.0	0.825	23.0 ± 1.1	20.8 ± 1.0	0.137
Sugars	2.9 ± 0.1	2.9 ± 0.1	0.424	2.9 ± 0.1	2.8 ± 0.1	0.805
Beans	37.2 ± 1.3	38.4 ± 1.7	0.578	36.3 ± 1.8	34.6 ± 1.5	0.464
Vegetables	156.6 ± 3.8	144.5 ± 4.9	0.052	156.8 ± 6.3	147.3 ± 5.6	0.261
Fruit	115.3 ± 4.1	119.3 ± 5.3	0.555	123.6 ± 6.1	119.0 ± 5.4	0.571
Fish	40.2 ± 1.2	38.7 ± 1.5	0.442	40.6 ± 1.7	40.3 ± 1.5	0.916
Meat	45.4 ± 1.1	46.5 ± 1.4	0.560	44.3 ± 1.5	45.9 ± 1.3	0.421
Eggs	18.7 ± 0.7	19.5 ± 0.9	0.507	19.3 ± 1.1	20.0 ± 1.0	0.671
Dairy	246.1 ± 7.4	240.7 ± 9.6	0.653	231.9 ± 10.3	233.0 ± 9.1	0.939
Fats and oils	8.6 ± 0.2	8.9 ± 0.2	0.374	8.9 ± 0.2	9.0 ± 0.2	0.839
Confectionery	34.0 ± 1.0	33.9 ± 1.3	0.964	32.9 ± 1.5	34.8 ± 1.3	0.338
Beverage other than alcohol	206.6 ± 8.1	212.5 ± 10.4	0.712	186.9 ± 12.0	216.6 ± 10.6	0.065

^a^K-6 score ≥10. ^b^*P* value was calculated as ANCOVA with the child's sex and age as covariates. ^c^Each food intake was energy adjusted using the residual model.

**Table 5:** Energy and food intake of three-year-old children of mothers, with and without mood disorders, and of those with and without friends with whom they can talk about childbirth and childcare.

(g)	Without mood disorder (*n* = 559)		*P* value^b^	With mood disorder (*n* = 361)		*P* value^b^
	With support	Without support		With support	Without support	
	(*n* = 357)	(*n* = 202)		(*n* = 158)	(*n* = 203)	
	(Mean ± SE)	(Mean ± SE)		(Mean ± SE)	(Mean ± SE)	
Energy (kcal)	1406.0 ± 18.2	1387.2 ± 24.2	0.537	1440.6 ± 25.6	1365.7 ± 22.6	0.029
Cereals	308.5 ± 4.2	302.3 ± 5.6	0.373	295.2 ± 6.1	323.2 ± 5.4	0.001
Potatoes and starches	22.1 ± 0.8	24.1 ± 1.0	0.115	21.0 ± 1.1	22.3 ± 1.0	0.407
Sugars	3.0 ± 0.1	2.8 ± 0.1	0.012	2.9 ± 0.1	2.8 ± 0.1	0.331
Beans	38.2 ± 1.3	36.6 ± 1.7	0.477	35.7 ± 1.8	35.0 ± 1.5	0.766
Vegetables	156.9 ± 3.8	143.5 ± 5.0	0.033	161.0 ± 6.3	144.1 ± 5.6	0.046
Fruit	120.9 ± 4.1	109.7 ± 5.4	0.099	130.5 ± 6.0	113.7 ± 5.3	0.037
Fish	40.0 ± 1.1	39.0 ± 1.5	0.614	42.7 ± 1.7	38.6 ± 1.5	0.070
Meat	45.4 ± 1.1	46.6 ± 1.5	0.521	45.8 ± 1.5	44.6 ± 1.3	0.552
Eggs	18.8 ± 0.7	19.3 ± 1.0	0.724	19.3 ± 1.1	20.0 ± 1.0	0.671
Dairy	240.4 ± 7.3	250.6 ± 9.8	0.402	235.4 ± 10.3	230.3 ± 9.1	0.709
Fats and oils	8.5 ± 0.2	9.1 ± 0.2	0.032	8.9 ± 0.2	9.0 ± 0.2	0.681
Confectionery	34.3 ± 1.0	33.3 ± 1.3	0.573	35.5 ± 1.5	32.8 ± 1.3	0.169
Beverage other than alcohol	214.9 ± 8.0	197.2 ± 10.6	0.184	210.9 ± 12.1	197.9 ± 10.7	0.422

^a^K-6 score ≥10. ^b^*P* value was calculated as ANCOVA with the child's sex and age as covariates. ^c^Each food intake was energy adjusted using the residual model.

## Data Availability

The restrictions by ethics committee at National Center for Child Health and Development prohibit the authors from making the minimal data set publicly available. Data are available from the Boshi Cohort Central Office located in the National Center for Child Health and Development, for researchers who meet the criteria for access to confidential data. Contact information for Boshi Cohort Central is boshi-cohort@ncchd.go.jp.

## Supplementary Materials

Supplementary Materials Supplementary Table 1: energy and food intake of three-year-old children of mothers who work full-time and with mood disorders and of those who are not full-time workers but have mood disorders, both those with and without friends with whom they can talk about childbirth and childcare. Supplementary Table 2: energy and food intake of three-year-old children of mothers with and without postpartum depression (PDa), for those with mood disorders (MDa), and of those with and without friends with whom they can talk about childbirth and childcare.
