# Supplementary material for: Relationship between Maternal Mood Disorders and Dietary Intake of 3-Year-Olds
Source: J Nutr Metab. 2021 Dec 17;2021:5597836. doi: 10.1155/2021/5597836 (PMC8709751; doi:10.1155/2021/5597836)
Supplement: Supplementary Materials — Supplementary Table 1: energy and food intake of three-year-old children of mothers who work full-time and with mood disorders and of those who are not full-time workers but have mood disorders, both those with and without friends with whom they can talk about childbirth and childcare. Supplementary Table 2: energy and food intake of three-year-old children of mothers with and without postpartum depression (PDa), for those with mood disorders (MDa), and of those with and without friends with whom they can talk about childbirth and childcare. [file 5597836.f1.docx]

Supplementary table 1. Energy and food intake of three-year-old children of mothers who work full-tme and with mood disorders, and of those who are not work full-time workers but have mood disorders, both those with and without friends with whom they can talk about childbirth and childcare.

|  |  | Part-time worker and unemployed (n = 314) | | | | | |  |  | Full-time worker (n = 43) | | | | | |  |
| --- | --- | --- | --- | --- | --- | --- | --- | --- | --- | --- | --- | --- | --- | --- | --- | --- |
|  |  | With support | | | Without support | | | *P* value^b^ |  | With support | | | Without support | | | *P* value^b^ |
| (g) |  | (n = 136) | | | (n = 178) | | |  |  | (n = 21) | | | (n = 22) | | |  |
|  |  | (mean ± SE) | | | (mean ± SE) | | |  |  | (mean ± SE) | | | (mean ± SE) | | |  |
| Energy (kcal) |  | 1427.2 | ± | 27.1 | 1351.1 | ± | 23.7 | 0.036 |  | 1550.1 | ± | 75.6 | 1460.6 | ± | 73.8 | 0.403 |
| Cereals |  | 292.1 | ± | 6.6 | 327.0 | ± | 5.8 | <0.0001 |  | 318.1 | ± | 15.1 | 296.7 | ± | 14.7 | 0.319 |
| Potatoes and starches |  | 21.2 | ± | 1.2 | 22.2 | ± | 1.1 | 0.527 |  | 20.2 | ± | 3.2 | 22.7 | ± | 3.1 | 0.581 |
| Sugars |  | 2.9 | ± | 0.1 | 2.8 | ± | 0.1 | 0.165 |  | 2.7 | ± | 0.2 | 2.7 | ± | 0.2 | 0.868 |
| Beans |  | 35.6 | ± | 1.9 | 34.9 | ± | 1.6 | 0.790 |  | 35.8 | ± | 5.5 | 38.4 | ± | 5.4 | 0.741 |
| Vegetables |  | 160.0 | ± | 6.9 | 144.2 | ± | 6.0 | 0.083 |  | 168.6 | ± | 17.5 | 136.3 | ± | 17.1 | 0.196 |
| Fruit |  | 133.0 | ± | 6.6 | 110.5 | ± | 5.8 | 0.011 |  | 116.9 | ± | 14.7 | 130.1 | ± | 14.4 | 0.524 |
| Fish |  | 43.7 | ± | 1.8 | 38.8 | ± | 1.6 | 0.046 |  | 37.0 | ± | 5.0 | 38.8 | ± | 4.8 | 0.792 |
| Meat |  | 46.5 | ± | 1.7 | 44.1 | ± | 1.4 | 0.277 |  | 41.5 | ± | 3.6 | 49.3 | ± | 3.5 | 0.126 |
| Eggs |  | 19.3 | ± | 1.2 | 19.6 | ± | 1.0 | 0.852 |  | 19.9 | ± | 3.0 | 21.9 | ± | 2.9 | 0.642 |
| Dairy |  | 237.6 | ± | 11.2 | 228.0 | ± | 9.8 | 0.518 |  | 219.1 | ± | 22.8 | 241.7 | ± | 22.3 | 0.485 |
| Fats and Oils |  | 8.8 | ± | 0.3 | 9.1 | ± | 0.2 | 0.461 |  | 9.2 | ± | 0.7 | 8.9 | ± | 0.7 | 0.793 |
| Confectionery |  | 35.6 | ± | 1.6 | 32.1 | ± | 1.4 | 0.093 |  | 34.3 | ± | 4.2 | 37.5 | ± | 4.1 | 0.601 |
| Beverage other than alcohol |  | 210.7 | ± | 13.1 | 197.6 | ± | 11.4 | 0.453 |  | 215.7 | ± | 32.7 | 217.8 | ± | 32.0 | 0.964 |

a K-6 score ≧10

b P value was calculated as ANCOVA with the child's sex and age as covariates.

c Each food intake was energy adjusted using residual model.

Analysis excluded 4 respondents who did not provide their employment status.

Supplementary table 2. Energy and food intake of three-year-old children of mothers with and without postpartum depression (PDa), for those with mood disorders (MDa), and of those, both those with and without friends with whom they can talk about childbirth and childcare

|  |  | Without postpartum depression (n = 267) | | | | | |  |  | With postpartum depression (n = 94) | | | | | |  |
| --- | --- | --- | --- | --- | --- | --- | --- | --- | --- | --- | --- | --- | --- | --- | --- | --- |
|  |  | with support | | | without support | | | *P* value^b^ |  | with support | | | without support | | | *P* value^b^ |
| (g) |  | (n = 131) | | | (n = 136) | | |  |  | (n = 27) | | | (n = 67) | | |  |
|  |  | (mean ± SE) | | | (mean ± SE) | | |  |  | (mean ± SE) | | | (mean ± SE) | | |  |
| Energy (kcal) |  | 1428.3 | ± | 28.1 | 1385.2 | ± | 27.5 | 0.275 |  | 1501.8 | ± | 63.1 | 1325.6 | ± | 39.9 | 0.021 |
| Cereals |  | 297.2 | ± | 6.7 | 323.2 | ± | 6.5 | 0.006 |  | 284.8 | ± | 15.5 | 323.3 | ± | 9.8 | 0.039 |
| Potatoes and starches |  | 20.9 | ± | 1.1 | 21.4 | ± | 1.1 | 0.761 |  | 21.6 | ± | 3.3 | 24.0 | ± | 2.1 | 0.537 |
| Sugars |  | 2.9 | ± | 0.1 | 2.8 | ± | 0.1 | 0.279 |  | 2.8 | ± | 0.2 | 2.8 | ± | 0.1 | 0.773 |
| Beans |  | 35.7 | ± | 2.0 | 35.6 | ± | 1.9 | 0.974 |  | 35.7 | ± | 4.0 | 34.0 | ± | 2.5 | 0.723 |
| Vegetables |  | 155.8 | ± | 6.6 | 140.3 | ± | 6.5 | 0.095 |  | 189.2 | ± | 17.2 | 150.5 | ± | 10.9 | 0.061 |
| Fruit |  | 127.2 | ± | 6.5 | 113.4 | ± | 6.3 | 0.131 |  | 143.6 | ± | 15.9 | 115.5 | ± | 10.1 | 0.139 |
| Fish |  | 43.2 | ± | 1.9 | 37.6 | ± | 1.9 | 0.035 |  | 41.3 | ± | 4.0 | 40.4 | ± | 2.5 | 0.847 |
| Meat |  | 45.2 | ± | 1.6 | 44.3 | ± | 1.6 | 0.695 |  | 48.5 | ± | 3.9 | 45.7 | ± | 2.5 | 0.557 |
| Eggs |  | 19.8 | ± | 1.2 | 21.4 | ± | 1.2 | 0.360 |  | 17.6 | ± | 2.5 | 16.9 | ± | 1.6 | 0.818 |
| Dairy |  | 231.6 | ± | 11.0 | 224.2 | ± | 10.8 | 0.631 |  | 256.4 | ± | 27.2 | 241.6 | ± | 17.2 | 0.649 |
| Fats and Oils |  | 8.9 | ± | 0.3 | 8.8 | ± | 0.3 | 0.973 |  | 9.3 | ± | 0.6 | 9.4 | ± | 0.4 | 0.905 |
| Confectionery |  | 35.9 | ± | 1.6 | 34.7 | ± | 1.6 | 0.607 |  | 33.4 | ± | 3.5 | 29.0 | ± | 2.2 | 0.300 |
| Beverage other than alcohol |  | 210.5 | ± | 13.4 | 200.9 | ± | 13.2 | 0.614 |  | 217.0 | ± | 29.0 | 190.2 | ± | 18.3 | 0.436 |

a MD, k6 score ≧10, PD, EPDS score ≧9

b P value was calculated as ANCOVA with the child's sex and age as covariates.

c Each food intake was energy adjusted using residual model.
